# Schisandrin A inhibits dengue viral replication via upregulating antiviral interferon responses through STAT signaling pathway

**DOI:** 10.1038/srep45171

**Published:** 2017-03-24

**Authors:** Jung-Sheng Yu, Yu-Hsuan Wu, Chin-Kai Tseng, Chun-Kuang Lin, Yao-Chin Hsu, Yen-Hsu Chen, Jin-Ching Lee

**Affiliations:** 1 Department of Chinese Medicine, Chi Mei Medical Center, Tainan 71004, Taiwan; 2 Graduate Institute of Integrated Medicine, China Medical University, Taichung 40402, Taiwan; 3 Institute of Basic Medical Sciences, College of Medicine, National Cheng Kung University, Tainan, Taiwan; 4 Center of Infectious Disease and Signaling Research, College of Medicine, National Cheng Kung University, Tainan, Taiwan; 5 Doctoral Degree Program in Marine Biotechnology, College of Marine Sciences, National Sun Yat-Sen University, Kaohsiung, Taiwan; 6 Division of Infectious Diseases, Department of Internal Medicine, Kaohsiung Medical University, Hospital, Kaohsiung Medical University, Kaohsiung, Taiwan; 7 School of Medicine, College of Medicine, Kaohsiung Medical University, Kaohsiung, Taiwan; 8 Department of Laboratory Medicine, Kaohsiung Medical University Hospital, Kaohsiung Medical University, Kaohsiung, Taiwan; 9 Center for Dengue Fever Control and Research, Kaohsiung Medical University, Kaohsiung, Taiwan; 10 Department of Biotechnology, College of Life Science, Kaohsiung Medical University, Kaohsiung, Taiwan; 11 Graduate Institute of Natural Products, College of Pharmacy, Kaohsiung Medical University, Kaohsiung, Taiwan; 12 Research Center for Natural Products and Drug Development, Kaohsiung Medical University, Kaohsiung, Taiwan; 13 Graduate Institute of Medicine, College of Medicine, Kaohsiung Medical University, Kaohsiung, Taiwan

## Abstract

Dengue virus (DENV) infects 400 million people worldwide annually. Infection of more than one serotype of DENV highly corresponds to dengue hemorrhagic fever and dengue shock syndrome, which are the leading causes of high mortality. Due to lack of effective vaccines and unavailable therapies against DENV, discovery of anti-DENV agents is urgently needed. We first characterize that Schisandrin A can inhibit the replication of four serotypes of DENV in a concentration- and time-dependent manner, with an effective half-maximal effective concentration 50% (EC_50_) value of 28.1 ± 0.42 μM against DENV serotype type 2 without significant cytotoxicity. Furthermore, schisandrin A can effectively protect mice from DENV infection by reducing disease symptoms and mortality of DENV-infected mice. We demonstrate that STAT1/2-mediated antiviral interferon responses contribute to the action of schisandrin A against DENV replication. Schisandrin A represents a potential antiviral agent to block DENV replication *in vitro* and *in vivo*. In conclusion, stimulation of STAT1/2-mediated antiviral interferon responses is a promising strategy to develop antiviral drug.

Dengue virus (DENV) is an arthropod-borne pathogen of a human viral disease, which infects 400 million people in the world, and 2.5 billion people are at risk of infection in tropical and subtropical areas^1^. DENV belongs to the Flavivirus genus of the Flaviviridae family. The genome of DENV is a positive sense of 11-kb single-stranded RNA encoding a single polyprotein^2^. The polyprotein is processed by viral and host proteases, resulting in the maturation of three structural proteins (C, prM, and E) and seven nonstructural proteins (NS1, NS2A, NS2B, NS3, NS4A, NS4B, and NS5)^3^. DENV is classified into four serotypes (DENV 1–4)^4^, which is of importance with regard to the clinical manifestations ranging from dengue fever (DF) to dengue hemorrhagic fever (DHF) and dengue shock syndrome (DSS)^5^,^6^. Infection with more than one serotype of DENV could cause high risk of DHF and DSS, leading to tens of thousands of deaths annually^7^,^8^. Today, because of lack of approved drugs or effective vaccines against DENV infection, it is of utmost importance to find new therapeutics to treat the disease.

Type I interferon (IFN-I) pathway is an important innate immune response to protect the host from pathogen invasion^7^,^9^. The binding of IFN-I cytokines, such as IFN-α and IFN-β, and cell surface type I IFN-alpha receptor (IFNAR) activates phosphorylation of Janus kinases 1 (JAK1) and tyrosine kinase 2 (Tyk2), which leads to activation of the signal transducer and activator of transcription factors 1 and 2 (STAT1 and STAT2) via tyrosine phosphorylation^10^. Subsequently, phosphorylated STAT1–STAT2 heterodimer binds to IFN-regulated gene 9 and form a transcriptional complex of IFN-stimulated gene factor 3 (ISGF3). ISGF3 then translocates into the nucleus and binds to the IFN- stimulated response element (ISRE) to trigger the expression of several antiviral IFN-stimulated genes (ISGs), including 2′-5′-oligoadenylate synthetase 1 (OAS1), OAS2, OAS3, and protein kinase R (PKR), which ultimately collapses different stages of virus replication^9^,^11^,^12^. In case of DENV infection, NS2B/3 protease subverts IFN activation by targeting MITA protein, NS4B interferes IFN pathway by blocking STAT1 phosphorylation, and NS5 blocks JAK-STAT2 pathway by degradation of STAT2 protein to prevent antiviral gene expression^9^,^13^,^14^. Therefore, IFN-mediated antiviral responses display a critical role in the prevention of DENV infection and DENV-related pathogenesis.

*Schisandra chinensis* (Turcz.) Baill. (*S. chinensis*) is a widely used herbal medicine, and its fruit is frequently used as a sedative, analgesic, and antipyretic and to treat hepatitis, myocardial disorders, and hyperlipidemia^15^,^16^. In addition, the extract of *S. chinensis* has also been used to treat neurodegenerative diseases, such as Alzheimer’s and Parkinson’s disease^17^. Nine major bioactive ingredients within *S. chinensis* were identified, including schisandrol A, schisandrol B, angeloylgomisin H, gomisin G, schisantherin A, schisanhenol, schisandrin A, schisandrin B, and schisandrin C^16^. Current reports demonstrated that these ingredients of *S. chinensis* possess several biological activities, such as antioxidant, antitumor, anti-inflammatory, and immunoregulatory activities^15^,^18^,^19^,^20^,^21^,^22^,^23^, and schisandrin A and schisandrin B exhibit antiviral activity against HIV^24^. Here, we investigated whether schisandrin A, schisandrin B, or schisandrin C could exhibit anti-DENV activity *in vitro* and *in vivo* and further investigated the molecular mechanism by which the effective ingredient inhibits DENV replication.

## Results

### Schisandrin A inhibits DENV RNA replication and protein synthesis

**Figure 1:**
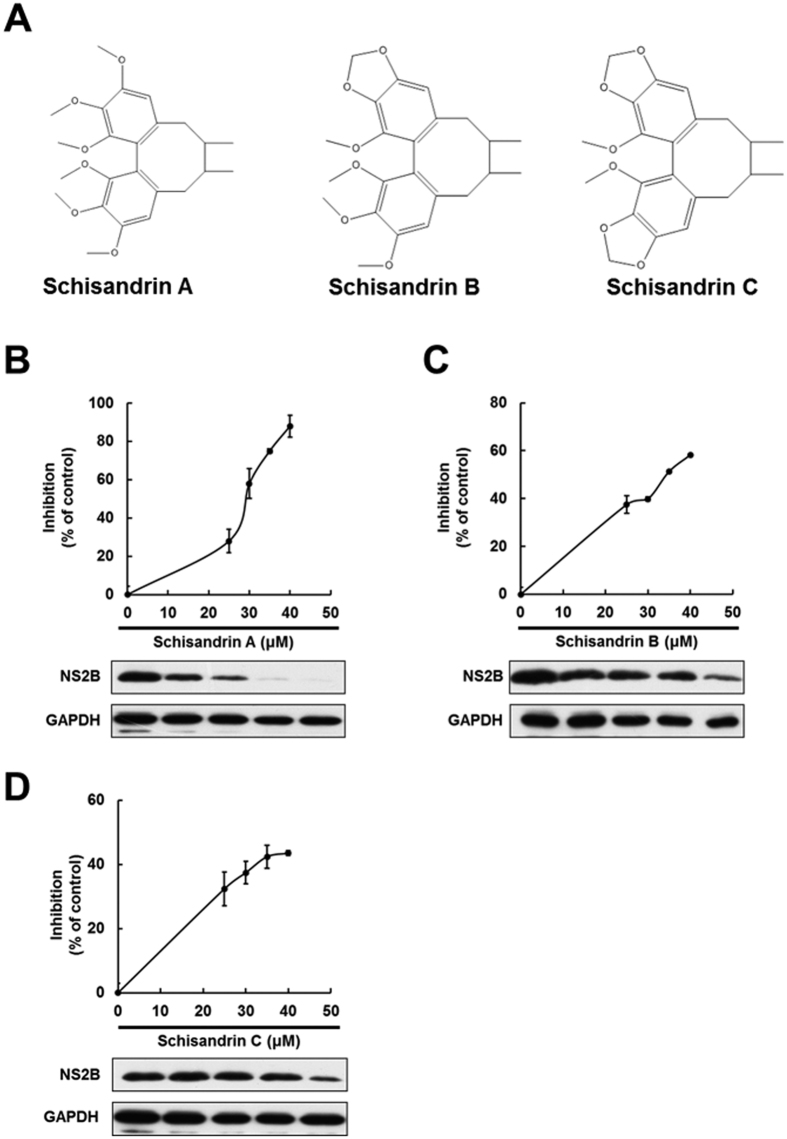
Schisandrin A efficiently inhibits DENV replication in a dose-dependent manner. (**A**) Structure of schisandrin A, schisandrin B, and schisandrin C. (**B**–**D**) Schisandrin A efficiently inhibits DENV RNA replication and protein synthesis. Huh-7 cells were infected with DENV for 2 h, followed by 0.1% of DMSO (dose 0, a negative control), 25, 30, 35, and 40 μM of schisandrin A, schisandrin B, or schisandrin C treatment for 3 days. The DENV RNA and protein levels were analyzed by RT-qPCR and Western blotting, respectively. For RT-qPCR, the DENV RNA level was normalized by the cellular *gapdh* mRNA level. The relative DENV RNA of each sample was presented as the % change compared to schisandrin A-untreated control (100%). For Western blotting, GAPDH served as an equal loading control. Error bars denote the means ± SD of five independent experiments (N = 5). *P < 0.05.

**Figure 2:**
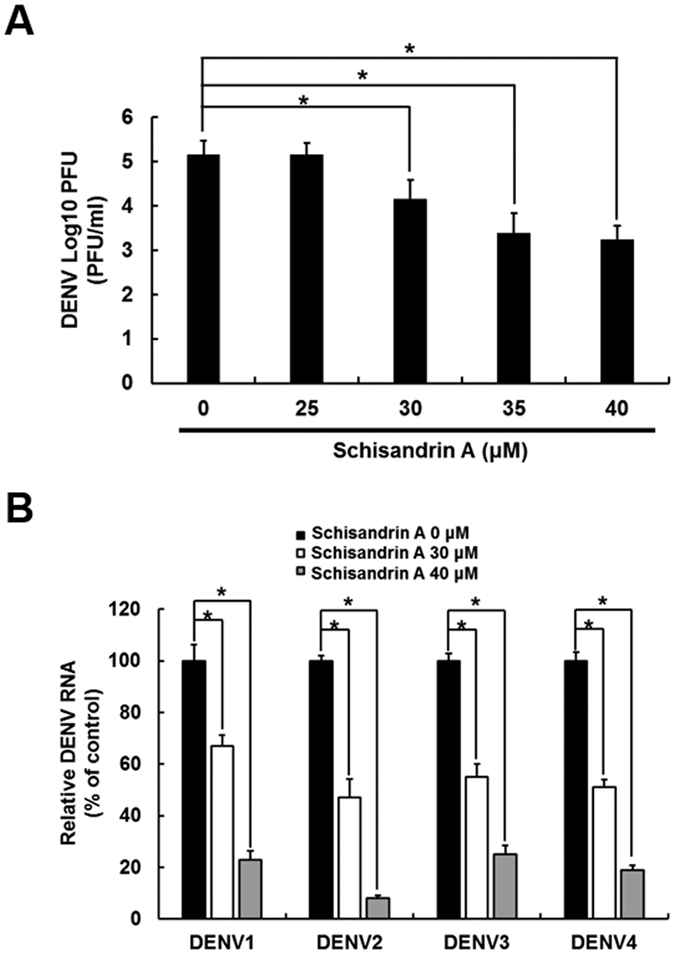
Schisandrin A inhibits replication of four serotypes of DENV. (**A**) Schisandrin A inhibits DENV titer. Huh-7 cells were infected by DENV at an MOI of 0.1 for 2 h and treated by 0.1% of DMSO (dose 0, a negative control), 25, 30, 35 and 40 μM schisandrin A. After 3 days treatment, the supernatant was collected, and the DENV titer was determined by plague assay. (**B**) Huh-7 cells were infected with the four DENV serotypes at an MOI of 0.1 for 2 h, followed by 0 (black bars), 30 (white bars), and 40 μM (gray bars) of schisandrin A treatment. The DENV RNA level was normalized by the cellular *gapdh* mRNA level in RT-qPCR. The relative DENV RNA of each sample was presented as the % change compared to schisandrin A-untreated control (100%). Error bars denote the means ± SD of five independent experiments (N = 5). *P < 0.05.

The DENV-infected Huh-7 cells were treated with individual schisandrin derivatives at the indicated concentrations for 3 days. DENV RNA and protein levels were analyzed by RT-qPCR and Western blotting, respectively, in which the amount of NS2B represented the DENV protein synthesis level. As shown in Fig. 1B, schisandrin A effectively reduced DENV RNA and protein levels compared with schisandrin A-untreated cells. In contrast. schisandrin B and schisandrin C exerted lower inhibitory effect on DENV RNA and protein levels than that of schisandrin A-treated cells (Fig. 1C and D). The half-maximal effective concentration (EC_50_) values of schisandrin A, schisandrin B, and schisandrin C were determined as 28.1 ± 0.42 μM, 34.0 ± 0.95 μM, and 42.6 ± 3.48 μM, respectively. No significant cytotoxicity was observed when the cells were exposed to effective antiviral concentration of each schisandrin derivative (Figure S1). Therefore, we selected schisandrin A as a potential inhibitor for subsequent studies. We first examined whether schisandrin A treatment could suppress viral titer of DENV, and the result showed that schisandrin A treatment decreased DENV titer (Fig. 2A). We further tested the antiviral effect of schisandrin A on the four DENV serotypes. The DENV-infected Huh-7 cells were treated with 30 or 40 μM of schisandrin A for 3 days. The RT-qPCR results revealed that schisandrin A can block the replication of the four serotypes of DENV in a concentration-dependent manner (Fig. 2B).

### Schisandrin A decreases the mortality of DENV-infected ICR suckling mice

**Figure 3:**
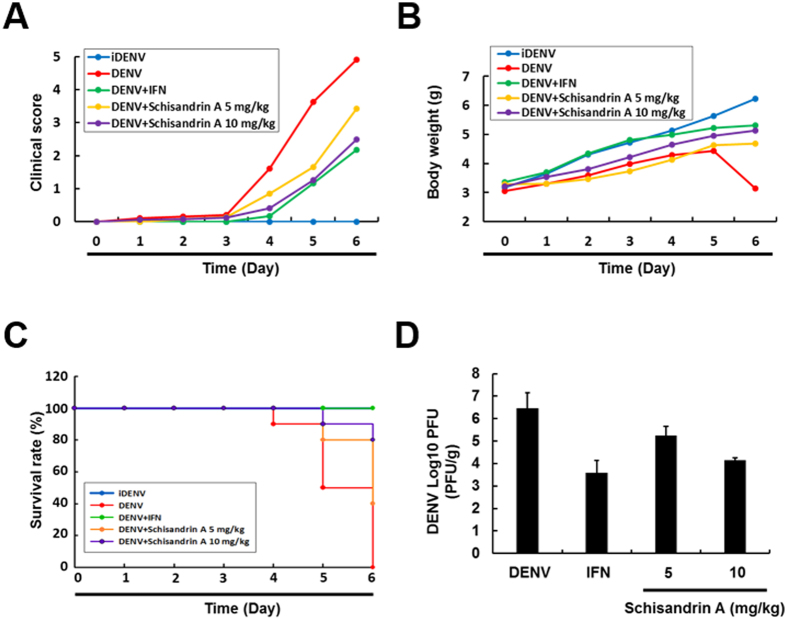
Schisandrin A protects ICR suckling mice from DENV infection. (**A**–**D**) Six-day-old ICR suckling mice were intracerebrally injected with heat-inactivated DENV (iDENV, blue line) or active DENV. DENV-infected mice received saline (DENV, blue line), 100 U/g IFN (DENV+IFN, green line), 5 (DENV+schisandrin A 5 mg/kg, yellow line) or 10 mg/kg (DENV+Schisandrin A 10 mg/kg, purple line) schisandrin A treatment every 2 days. Mice were sacrificed at 6 days postinfection, and the (**A**) clinical scores, (**B**) body weight, and (**C**) survival rates were measured daily. Disease severity was scored as follows: 0: no symptom, 1: slight losing weight and ruffled hair, 2: slowing of activity, 3: asthenia, 4: paralysis and mortally ill, and 5: death. (**D**) The brain tissue was collected to analyze the virus titer by plaque-forming assay. Each group included ten mice. Error bars denote the means ± SD. *P < 0.05.

To further examine the anti-DENV activity of schisandrin A *in vivo*, 6-day-old ICR suckling mice were intracerebrally injected with active DENV or heat-inactivated DENV (iDENV), in which injection of iDENV served as a negative control, and injection of 100 U/g IFN served as a positive control. The DENV-infected mice were administered either saline or schisandrin A injection at 1, 3, and 5 days postinfection (dpi). The clinical score, body weight, and the survival rate were daily monitored. The brain tissue was collected to analyze the virus titer using plaque assay at 6 dpi. As shown in Fig. 3A, the DENV-infected mice treated with schisandrin A showed a lower clinical score than that of mice treated with saline and the iDENV-infected control mice. The body weights of DENV-infected mice receiving schisandrin A treatment were recovered, compared to those of iDENV-infected or DENV-infected mice at 6 dpi (Fig. 3B). The survival rate of DENV-infected mice treated with schisandrin A reached 80%, compared to that of DENV-infected mice with saline treatment at 6 dpi (Fig. 3C). An approximate two log decrease in the viral titer in the brain of DENV-infected mice treated with schisandrin A was measured, compared to that of DENV-infected mice without schisandrin A treatment (Fig. 3D).

### Schisandrin A induces antiviral IFN-I gene expression

**Figure 4:**
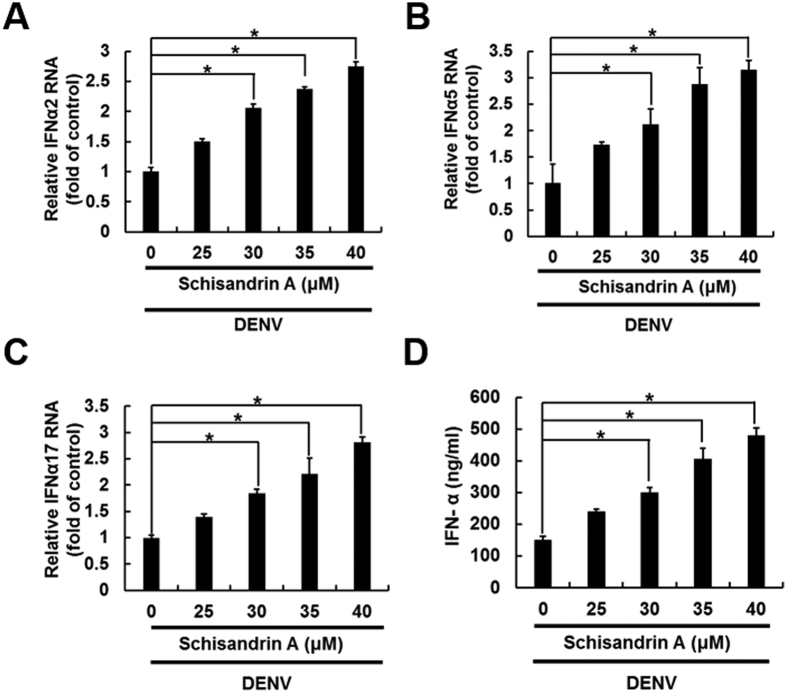
Schisandrin A induces antiviral IFN-α expression. (**A**–**D**) The DENV-infected Huh-7 cells were treated with 0.1% of DMSO (dose 0, a negative control), 25, 30, 35, and 40 μM of schisandrin A for 3 days. Total RNA was collected and the cellular (**A**) IFN-α-2, (**B**) IFN-α-5, and (**C**) IFN-α-17 mRNA levels were determined by RT-qPCR. The gene expression was normalized by the cellular *gapdh* mRNA level. (**D**) The supernatant was collected and the IFN-α protein level was analyzed using ELISA kit. Error bars denote the means ± SD of five independent experiments (N = 5). *P < 0.05.

IFN responses have been demonstrated to play an important role in resistance to viral infections. To evaluate whether antiviral IFN responses are involved in the anti-DENV activity of schisandrin A, the DENV-infected Huh-7 cells were treated with schisandrin A for 3 days, and the mRNA levels of IFN-alpha-2 (IFN-α-2), IFN-alpha-5 (IFN-α-5), and IFN-alpha-17 (IFN-α-17) were analyzed by RT-qPCR. As shown in Fig. 4A–C, schisandrin A significantly elevated DENV-reduced IFN-α gene expression in a dose-dependent manner. As expected, schisandrin A increased the amount of IFN-α protein production from DENV-infected Huh-7 cells in a concentration-dependent manner by ELISA analysis (Fig. 4D). Additionally, we also characterized that schisandrin A increased IFN-α-2, IFN-α-5 and IFN-α-17 expression in naïve Huh-7 cells (Figure S2), which indicated that schisandrin A exerts the inductive activity of antiviral IFN-I gene expression.

### Schisandrin A increased STAT1/2 phosphorylation for induction of antiviral IFN responses

**Figure 5:**
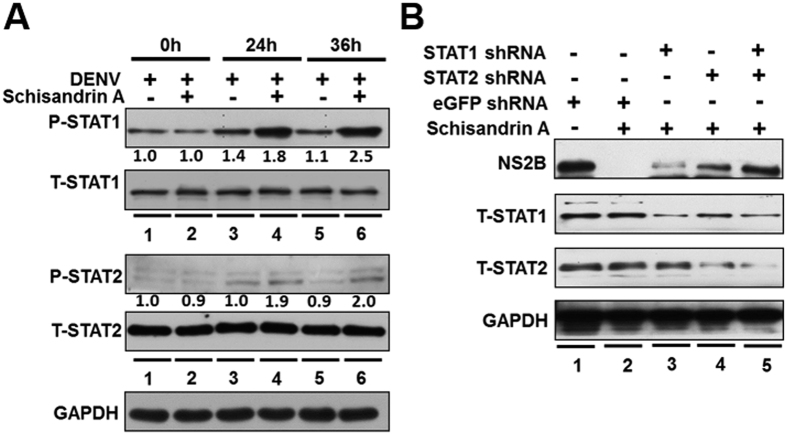
Schisandrin A stimulates STAT1 and STAT2 phosphorylation. (**A**) Accumulation of phosphorylated STAT1 and STAT2 levels upon schisandrin A. DENV-infected Huh-7 cells were treated with 0 or 40 μM of schisandrin A for 0, 24, or 36 h. STAT1 and STAT 2 phosphorylation (P-STAT1 and P-STAT2) was analyzed by Western blotting with anti-phosphorylated STAT1 and STAT2 antibodies. The total STAT1 and STAT2 level (T-STAT1 and T-STAT2) were determined by anti-STAT1 and STAT2 antibodies. The band intensities were quantified by densitometry, and the fold-change that was relative to schisandrin A-untreated control (defined as 1) at different time points was normalized by GAPDH protein level. (**B**) STAT1 and STAT2 silencing restored the inhibitory effect of schisandrin A on DENV replication. Huh-7 cells were transfected with different amounts of shRNA against STAT1, STAT2 (0.5 μg), or eGFP (0.5 μg), followed by DENV infection and schisandrin A treatment. DENV, STAT1, and STAT 2 protein levels were analyzed by Western blotting.

**Figure 6:**
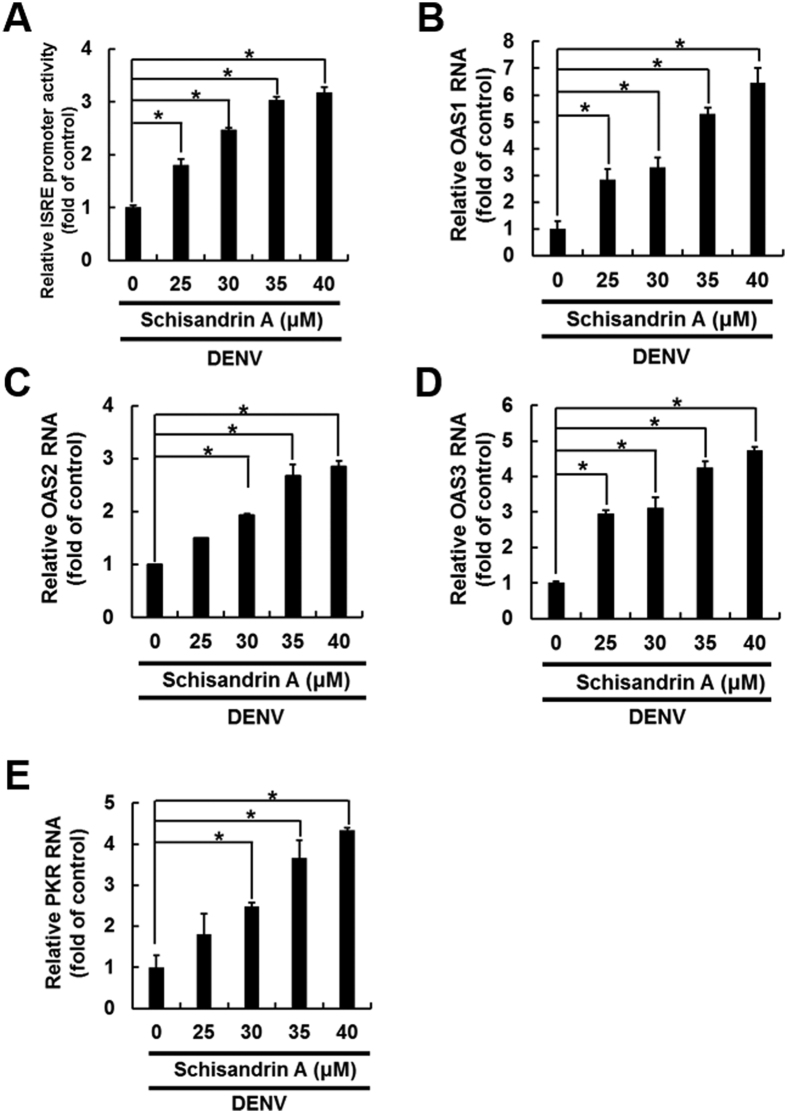
Schisandrin A induces ISRE activity and antiviral IFN response gene expression. (**A**) Induction of ISRE activity by schisandrin A. Huh-7 cells transiently expressing pISRE-Luc cells were infected with DENV, followed by treatment with 0.1% of DMSO (dose 0, a negative control), 25, 30, 35, and 40 μM of schisandrin A for 3 days. The luciferase activity was analyzed by Steady-GloLuciferase Assay System (Promega). (**B**–**E**) Induction of antiviral IFN responses by schisandrin A. DENV-infected Huh-7 cells were treated with schisandrin A at the indicated concentrations. After 3 days, the antiviral gene expression was analyzed by RT-qPCR using specific primers. The expression of genes was normalized by the cellular *gapdh* mRNA level. Error bars denote the means ± SD of five independent experiments (N = 5). *P < 0.05.

Phosphorylation of STAT1 and STAT2 is required for activation of IFN-mediated antiviral responses^9^. To further identify whether schisandrin A could mediate STAT activity for anti-DENV action, we examined the phosphorylation status of STAT1 and 2 in DENV-infected Huh-7 cells in the presence or absence of schisandrin A. The Western blotting results indicated that the amount of phospho-STAT1/2 level was time-dependently accumulated upon schisandrin A treatment (Fig. 5A, lanes 2, 4, and 6), with a 2- and 2.5-fold change relative to the schisandrin A-untreated control mice (Fig. 5A, lanes 1, 3, and 5), following quantification by densitometric analysis. To further clarify the role of STAT1/2-mediated signaling pathway in the anti-DENV activity of schisandrin A, we silenced STAT1/2 expression using specific shRNAs against STAT1 and STAT2, respectively, and DENV protein synthesis was measured by Western blotting. As shown in Fig. 5B, downregulation of STAT1/2 expression significantly restored DENV replication (upper panel, lanes 3–5) compared to nonspecific eGFP shRNA-transfected Huh-7 cells with or without schisandrin A treatment (lanes 1 and 2). To investigate the STAT1/2-dependent stimulation on antiviral IFN responses, Huh-7 cells were transiently transfected with pISRE-Luc reporter plasmid carrying IFN-stimulated response element (ISRE)-driven firefly luciferase in the presence of DENV infection and schisandrin A treatment for 3 days. As shown in Fig. 6A, the ISRE promoter activity was significantly increased with schisandrin A treatment. We subsequently examined the expression of IFN-based antiviral genes, including OAS1, OAS2, OAS3, and PKR, in DENV-infected Huh-7 cells with schisandrin A treatment. The RT-qPCR results showed that schisandrin A significantly stimulated the antiviral gene expression in a concentration-dependent manner (Fig. 6B–E).

### Schisandrin A inhibits DENV replication and stimulates IFN-mediated antiviral responses *in vivo*

**Figure 7:**
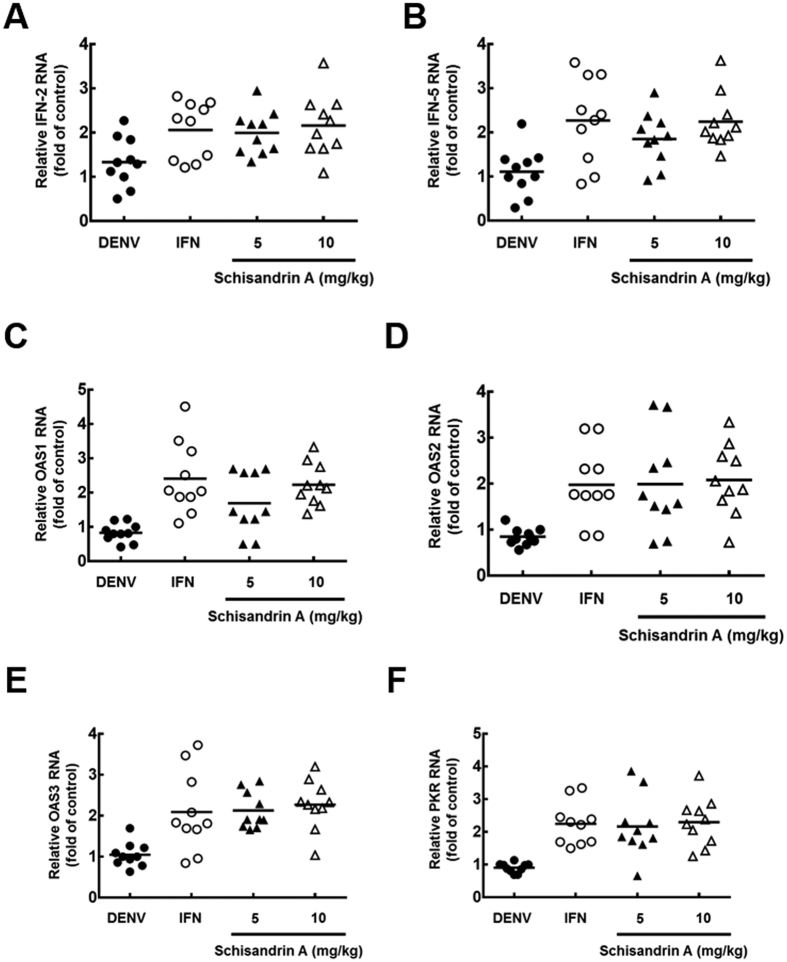
Schisandrin A inhibits DENV replication *in vivo* through modulating IFN-mediated antiviral responses. (**A**,**B**) DENV-infected suckling mice were sacrificed at 6 dpi, and then the brain tissue was collected to determine (**A**) IFN-α-2, (**B**) IFN-α-5, (**C**) OAS1, (**D**) OAS2, (**E**) OAS3, and (**F**) PKR expression levels by RT-qPCR using specific primers. Gene expression was normalized by the cellular *gapdh* mRNA level. Filled circles, open circles, filled triangles and filled triangles indicate schisandrin A-untreated, IFN-treated, 5 or 10 mg/kg of schisandrin A-treated mice, respectively. Each group included ten mice. *P < 0.05.

To confirm the mechanism by which schisandrin A inhibits DENV replication through the stimulation of IFN-mediated antiviral responses *in vivo*, we performed a DENV-infected ICR suckling mice model to measure IFN-α-2, IFN-α-5, and IFN-based antiviral gene expression in brain tissues by RT-qPCR analysis. As shown in Fig. 7A, both IFN-α-2 and IFN-α-5 RNA levels were induced by schisandrin A. Consistently, the RNA levels of IFN-based antiviral genes were also stimulated by schisandrin A due to IFN production (Fig. 7B).

## Discussion

**Figure 8:**
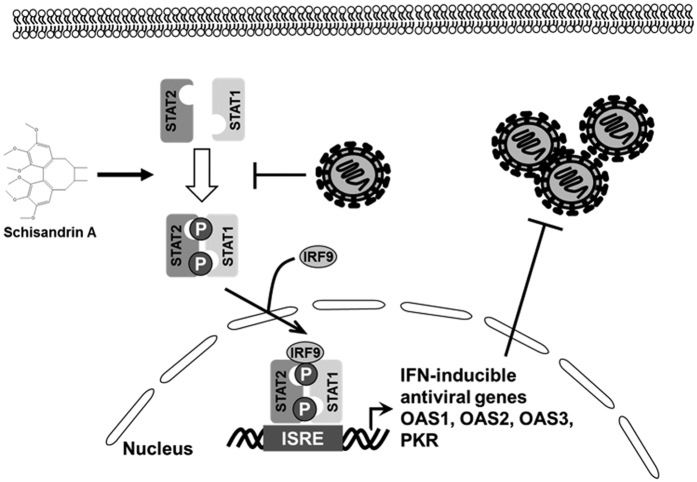
Model for schisandrin A against DENV replication. Schisandrin A enhances STAT1 and STAT2 phosphorylation and subsequently induces the antiviral interferon-stimulated gene expression for inhibition of DENV replication.

In the present study, we observed that schisandrin A treatment significantly induced IFN-α and its downstream antiviral ISG expression against DENV replication *in vitro* and *in vivo* (Figs 4, 6, 7 and S2). Furthermore, we verified that schisandrin A-mediated STAT1/2 phosphorylation is involved in IFN-based inhibition of DENV replication (Fig. 5) and proposed the action mechanism of schisandrin A against DENV replication (Fig. 8). These observations are consistent with the conclusion of Michael S. Diamond *et al*. that stimulation of antiviral IFN pathway is a promising strategy to disrupt DENV replication^25^. There are two signaling pathways to activate the host innate response against viral infection through production IFN-α and IFN-β; one is toll-like receptor (TLR)-mediated NF-κB, IFN regulatory factor-3 (IRF3), and IRF7 expression, and the other is RNA helicase retinoic acid-inducible gene I (RIG-I)-mediated mitochondrial antiviral signaling protein (MAVS) activation for activation of NF-κB and IRF3/7^26^,^27^. Further experiments will be carried out to investigate the correlation between schisandrin A and the upstream mediators of IFNs. To escape host antiviral immune responses and facilitate virus replication, viral proteins would interfere with antiviral IFN responses for an efficient virus production, such as human papilloma virus (HPV), human parainfluenza virus 2 (HPIV2), and West Nile virus (WNV)^28^,^29^,^30^. Current studies have demonstrated that enhanced ISG expression sufficiently suppresses DENV replication through IFN-induced transmembrane protein 2 and 3 (IFITM2 and 3), PKR, and OAS genes^7^,^12^. It would be noteworthy to further clarify how schisandrin A interrupts DENV protein-suppressed antiviral IFN pathway.

Infection with more than one serotype of DENV leads to high risk of DHF and DSS, which is the major cause of death among DENV-infected patients^5^. Previous studies reported that the plasma viremia in DHF/DSS patients is higher than that in DF patients, which indicates that reducing DENV viral level has the possibility to prevent or lessen the chances of patients progressing to DHF/DSS^7^,^8^. Our study demonstrated that schisandrin A can inhibit the four serotypes of DENV (Fig. 2), indicating that schisandrin A not only is a potential anti-DENV agent but also provides a capable therapy to prevent DHF/DSS. Previously, the extract of *S. chinensis* has been used in clinical trials to treat HCV infection, and no obvious side effects were observed^31^. In the present study, we identified that schisandrin A could protect ICR suckling mice from death due to DENV infection through the upregulation of IFN antiviral responses *in vitro* and *in vivo* (Figs 4 and 7), which offers the possibility of advancing the plant-derived natural product to clinical use against DENV infection and severe DENV-induced diseases.

In conclusion, we identified schisandrin A as a potential antiviral agent against DENV *in vitro* and *in vivo*. We further clarified the mechanism by which schisandrin A increased IFN expression and activated JAK-STAT pathway to trigger antiviral innate responses against DENV replication.

## Materials and Methods

### Ethics statement and experimental animals

Six-days-old ICR suckling mice were used in this study and beeder mice of the ICR strain were obtained from BioLasco Taiwan Co. Ltd. All animal studies were conducted in specific pathogen-free conditions and methods were carried out in accordance with the Guide for the Care and Use of Laboratory Animals. The experimental protocol were approved by the Animal Research Committee of Kaohsiung Medical University of Taiwan (IACUC, 102177) under the guidance of the Public Health Service (PHS) Policy on Humane Care and Use of Laboratory Animals. All mice received humane care and were fed with standard rodent chew and water ad libitum. Mice were acclimatized under a standard laboratory condition following the Animal Use Protocol of Kaohsiung Medical University for a week before experiment.

### Chemicals

Schisandrin A, schisandrin B, and schisandrin C (Fig. 1A) were purchased from Fusol-Material, Tainan, Taiwan. All these compounds were dissolved in dimethyl sulfoxide (DMSO) to establish stock solution of 50 mM and stored at −20 °C. All reactions were carried out in a final concentration of DMSO at 0.1%.

### Cells and virus

Huh-7 cells and BHK-21 cells were maintained in Dulbecco’s modified Eagle’s medium (DMEM) supplemented with 10% fetal bovine serum (FBS), 1% nonessential amino acids, and 1% antibiotic–antimycotic in 5% CO_2_ supplement at 37 °C. The C6/36 cells were maintained in RPMI 1640 medium supplemented with 10% FBS, 1% nonessential amino acids, 1% L-glutamine, 1% sodium pyruvate, and 1% antibiotic–antimycotic in 5% CO_2_ supplement at 37 °C. DENV-2 strain 16681 was kindly provided by Dr. Huey-Nan Wu, and the other types of DENV (DENV-1:DN8700828; DENV-3: DN8700829; DENV-4: S9201818) were obtained from the Centers for Disease Control, Department of Health, Taiwan. DENV was generated in the C6/36 mosquito cells^2^,^32^. Virus titer was determined by plaque assay.

### Plasmid construction

pISRE-Luc containing firefly luciferase under the control of an IFN-stimulated response element (ISRE) was used to measure IFN response-dependent transcriptional activity (Stratagene, Agilent Technologies, Palo Alto, CA, USA). STAT1 (NM_007315), STAT2 (NM_005419), and eGFP short hairpin RNA (shRNA) were purchased from the National RNAi Core Facility, Institute of Molecular Biology/Genomic Research Center, Academia Sinica, Taipei, Taiwan.

### Quantification of RNA level

Total cellular RNA was extracted by RNA Purification Kit (GMbiolab Co, Ltd., Taichung, Taiwan) following the manufacturer’s instructions. DENV RNA or cellular mRNA level was analyzed by quantitative real-time reverse-transcription polymerase chain reaction (RT-qPCR) with specific primers^2^. The gene expression level was normalized by the cellular glyceraldehyde-3-phosphate dehydrogenase (*gapdh*) mRNA level. Primers used in the study are listed in Table 1.

### Western blotting

The cells were lysed using RIPA lysis buffer and collected for Western blotting assay. The antibodies used in this study were anti-DENV NS2B antibody (1:3000; GeneTex, Inc, Irvine, CA), STAT1 antibody (1:3000; GeneTex), STAT2 antibody (GeneTex), phosphorylated STAT1 (Tyr701) antibody (1:1000; Cell Signaling Technology, Inc. Beverly, MA), phosphorylated STAT2 (Tyr690) antibody (1:1000; Cell Signaling), and anti-GAPDH antibody (1:10000; GeneTex), in which detection of GAPDH protein level served as an internal control^33^. DENV proteins are generated from proteolysis of single polypeptide translated from viral RNA, therefore each viral protein level can represent the DENV protein synthesis level. In our study, the NS2B served as an indicator of viral protein synthesis.

### Transfection

Huh-7 cells were seeded in 24-well plates and then transfected with the indicated plasmids by T-Pro™ transfection reagent (Ji-Feng Biotechnology Co., Ltd, Taipei, Taiwan) according to the manufacturer’s instructions.

### Measurement of luciferase activity

Luciferase reporter expression was analyzed by Steady-Glo Luciferase Assay System (Promega) according to the manufacturer’s instructions.

### Analysis of extracellular IFN-α protein level

Huh-7 cells were seeded in 24-well plates and then infected with DENV at an MOI of 0.2 for 2 h, followed by schisandrin A treatment. After 3 days, the supernatant was collected and IFN-α concentration was measured by human IFN-α ELISA kit (Life Science) according to the manufacturer’s protocol. Absorbance was detected at 450 nm using an Epoch microplate spectrophotometer (BioTek Instruments Inc., USA).

### Anti-DENV activity *in vivo*

Six-day-old ICR suckling mice were randomly divided into three groups (5 mice each group); Group 1 received 60 °C heat-inactivated DENV and saline treatment (iDENV); Group 2 received 2.5 × 10^5^ plaque-forming unit (PFU) of DENV and saline treatment (DENV); Group 3 receive 2.5 × 10^5^ PFU of DENV and 100 U/g of interferon treatment at 1, 3, and 5 days post infection (DENV+IFN); Group 4 received 2.5 × 10^5^ PFU of DENV and 5 mg/kg of schisandrin A treatment at 1, 3, and 5 days post infection (DENV+schisandrin A 5 mg/kg); and Group 5 received 2.5 × 10^5^ PFU of DENV and 10 mg/kg of schisandrin A treatment at 1, 3, and 5 days post infection (DENV+schisandrin A 10 mg/kg). The tested doses were chosen according to the study of Hu *et al*.^23^. After 6 days, the suckling mice were sacrificed by carbon dioxide euthanasia for determining the virus titer and virus RNA levels in brain tissues. To determine the virus titer, the brain tissues were weighed and homogenized in 0.5 ml RPMI 1640 medium supplemented with 2% FBS and then centrifuged at 8000 rpm for 15 min at 4 °C. Finally, the supernatants were collected and stored at −80 °C. To determine the DENV RNA levels, the brain tissues were weighed and homogenized in 0.5 ml TRIzol reagent (Invitrogen, Carlsbad, CA) and stored at −80 °C. The RNA samples were extracted according to the manufacturer’s instructions.

### Plaque assay

BHK-21 cells were seeded in 12-well plates and infected by serially diluted virus. After 2 h of incubation, the virus inoculum was refreshed with complete DMEM containing 0.8% methyl cellulose (Sigma-Aldrich, St. Louis, MO, USA) for 5 days. Then, the virus-infected cells were fixed and stained with crystal violet solution (1% crystal violet, 0.64% NaCl, and 2% formalin) for 1 h. The crystal violet solution was washed away and the virus titer was calculated.

### Statistical analysis

Data are expressed as mean ± standard deviations of at least five independent experiments (n ≥ 5). Statistical significance were determined using Student’s t test for differences between 2 data groups (drug-treated and -untreated cells). The n value indicates the number of experiments used. *P < 0.05 was considered to be significant.

## Additional Information

**How to cite this article**: Yu, J.-S. *et al*. Schisandrin A inhibits dengue viral replication via upregulating antiviral interferon responses through STAT signaling pathway. *Sci. Rep.* **7**, 45171; doi: 10.1038/srep45171 (2017).

**Publisher's note:** Springer Nature remains neutral with regard to jurisdictional claims in published maps and institutional affiliations.

## Supplementary Material

Supporting Information

## Figures and Tables

**Table 1:** Oligonucleotide sequences for real-time RT-PCR.

Oligonucleotide Name	Sequence 5′-3'
DENV gene oligonucleotide sequences	
Type 1- 5′ NS5	5′-CAGGTCAAACGCAGCTATTG
Type 1- 3′ NS5	5′-CCACTCCACTGAGTGAATTC
Type 2- 5′ NS5	5′-TGTATGCCGATGACACCGCA
Type 2- 3′ NS5	5′-TCTTTGCACACGGACCACCT
Type 3- 5′ NS5	5′-TCAGAACTAACGCAGCCATG
Type 3- 5′ NS5	5′-AGAGTTTTCACGCGAGAACC
Type 4- 5′ NS5	5′-AGATCAAACGCAGCCATAGG
Type 4- 5′ NS5	5′-CTTCCACTCCACTCCATGAA
Human gene oligonucleotide sequences	
5′ GAPDH	5′-GTCTTCACCACCATGGAGAA
3′ GAPDH	5′-ATGGCATGGACTGTGGTCAT
5′ OAS1	5′-CAAGCTTAAGAGCCTCATCC
3′ OAS1	5′-TGGGCTGTGTTGAAATGTGT
5′ OAS2	5′-ACAGCTGAAAGCCTTTTGGA
3′ OAS2	5′-GCATTAAAGGCAGGA AGCAC
5′ OAS3	5′-CACTGACATCCCAGACGATG
3′ OAS3	5′-GATCAGGCTCTTCAGCTTGG
5′ PKR	5′-ATGATGGAAAGCGAACAAGG
3′ PKR	5′-GAGATGATGCCATCCCGTAG
5′ IFN-alpha 2	5′-GCA AGT CAA GCT GCT CTG TG
3′ IFN-alpha 2	5′-GAT GGT TTC AGC CTT TTG GA
5′ IFN-alpha 5	5′-AGTTTGATGGCAACCAGTTC
3′ IFN-alpha 5	5′-TCAGAGGAGTGTCTTCCACT
5′ IFN-alpha 17	5′-AGG AGT TTG ATG GCA ACC AG
3′ IFN-alpha 17	5′-CAT CAG GGG AGT CTC TTC CA
Mice gene oligonucleotide sequences	
5′ GAPDH	5′-CCATGCCATCACTGCCACCC
3′ GAPDH	5′-GCCATGCCAGTGAGCTTCCC
5′ OAS1	5′-GGACCCCGCTGACCCAACAA
3′ OAS1	5′-CAACCTCCGTCGGCACCTCC
5′ OAS2	5′-GGTGGGTCTTCAGGGGTGCC
3′ OAS2	5′-GCAGCAGGTCCCAGATGGCA
5′ OAS3	5′-CCGTGCCTGGACTGAGCCTC
3′ OAS3	5′-GGCTGAGGTTTGGTGCCGGA
5′ PKR	5′-AGAGCCCGCCGAAAACTGCC
3′ PKR	5′-CGCTGTTAAACCTGGCGTCCA
5′ IFN-alpha 2	5′-TGACCTCCACCAGCAGCTCA
3′ IFN-alpha 2	5′-TCTGCTCTGACCACCTCCCA
5′ IFN-alpha 5	5′-CCATCCCTGTCCTGAGTGAGCT
3′ IFN-alpha 5	5′-AGATTCCTGCACCCCGACCT
