# Supplementary material for: Schisandrin A inhibits dengue viral replication via upregulating antiviral interferon responses through STAT signaling pathway
Source: Sci Rep. 2017 Mar 24;7:45171. doi: 10.1038/srep45171 (PMC5364541; doi:10.1038/srep45171)
Supplement: Supporting Information [file srep45171-s1.pdf]

Schisandrin A inhibits dengue viral replication via upregulating antiviral interferon responses through STAT signaling pathway

Jung-Sheng Yu[1,2¶], Yu-Hsuan Wu[3,4¶], Chin-Kai Tseng[3,4], Chun-Kuang Lin[5], Yao-Chin Hsu[1], Yen-Hsu Chen[6,7,8,9*], Jin-Ching Lee[10,11,12,13*]

[1]Department of Chinese Medicine, Chi Mei Medical Center, Tainan 71004, Taiwan.
[2]Chia Nan University of Pharmacy & Science, Tainan, 71710, Taiwan.
[3]Institute of Basic Medical Sciences, College of Medicine, National Cheng Kung University, Tainan, Taiwan.
[4]Center of Infectious Disease and Signaling Research, College of Medicine, National Cheng Kung University, Tainan, Taiwan.
[5] Doctoral Degree Program in Marine Biotechnology, College of Marine Sciences, National Sun Yat-Sen University, Kaohsiung, Taiwan.
[6]Division of Infectious Diseases, Department of Internal Medicine, Kaohsiung Medical University, Hospital, Kaohsiung Medical University, Kaohsiung, Taiwan.
[7]School of Medicine, College of Medicine, Kaohsiung Medical University, Kaohsiung, Taiwan.
[8]Department of Laboratory Medicine, Kaohsiung Medical University Hospital, Kaohsiung Medical University, Kaohsiung, Taiwan
[9]Center for Dengue Fever Control and Research, Kaohsiung Medical University, Kaohsiung, Taiwan.
[10]Department of Biotechnology, College of Life Science, Kaohsiung Medical University, Kaohsiung, Taiwan.
[11]Graduate Institute of Natural Products, College of Pharmacy, Kaohsiung Medical University, Kaohsiung, Taiwan.
[12]Research Center for Natural Products and Drug Development, Kaohsiung Medical University, Kaohsiung, Taiwan
[13]Graduate Institute of Medicine, College of Medicine, Kaohsiung Medical University, Kaohsiung, Taiwan
Running title: Anti-dengue virus activity of schisandrin A
***Corresponding authors:** Jin-Ching Lee and Yen-Hsu Chen

¶ Both authors contributed equally to this work.

Mail address: Department of Biotechnology, Kaohsiung Medical University, 100, Shih-Chuan 1st Road, San Ming District, 807 Kaohsiung City, Taiwan.
Phone: 886-7-312-1101 ext 2369 **Fax:** 886-7-312-5339
E-mail: jclee@kmu.edu.tw; infchen@gmail.com

Supporting information

Supplementary material and method

Cell cytotoxicity

Huh-7 cells were seeded in 96-well plates and treated with compounds at the indicated concentrations for 3 days. Cell viability was determined by the CellTiter 96 AQ_{ueous} One Solution Cell Proliferation Assay (Promega, Madison, WI) at 3 days posttreatment.

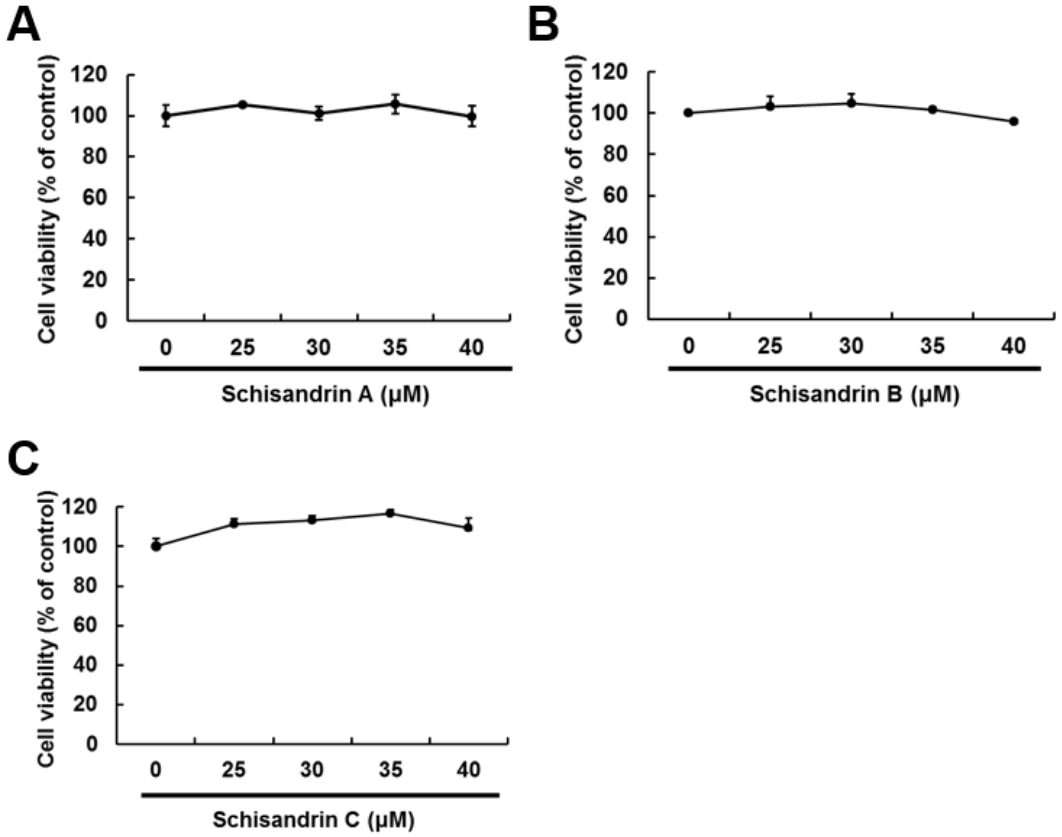

Fig S1. Cell viability of schisandrin A, schisandrin B, and schisandrin C.

(A–C) Huh-7 cells were treated with 0, 25, 30, 35, and 40 μM of schisandrin A, schisandrin B, and schisandrin C. After 3 days, cell cytotoxicity was analyzed by MTS assay. Error bars denote the means ± SD of five independent experiments (N=5). *P < 0.05.

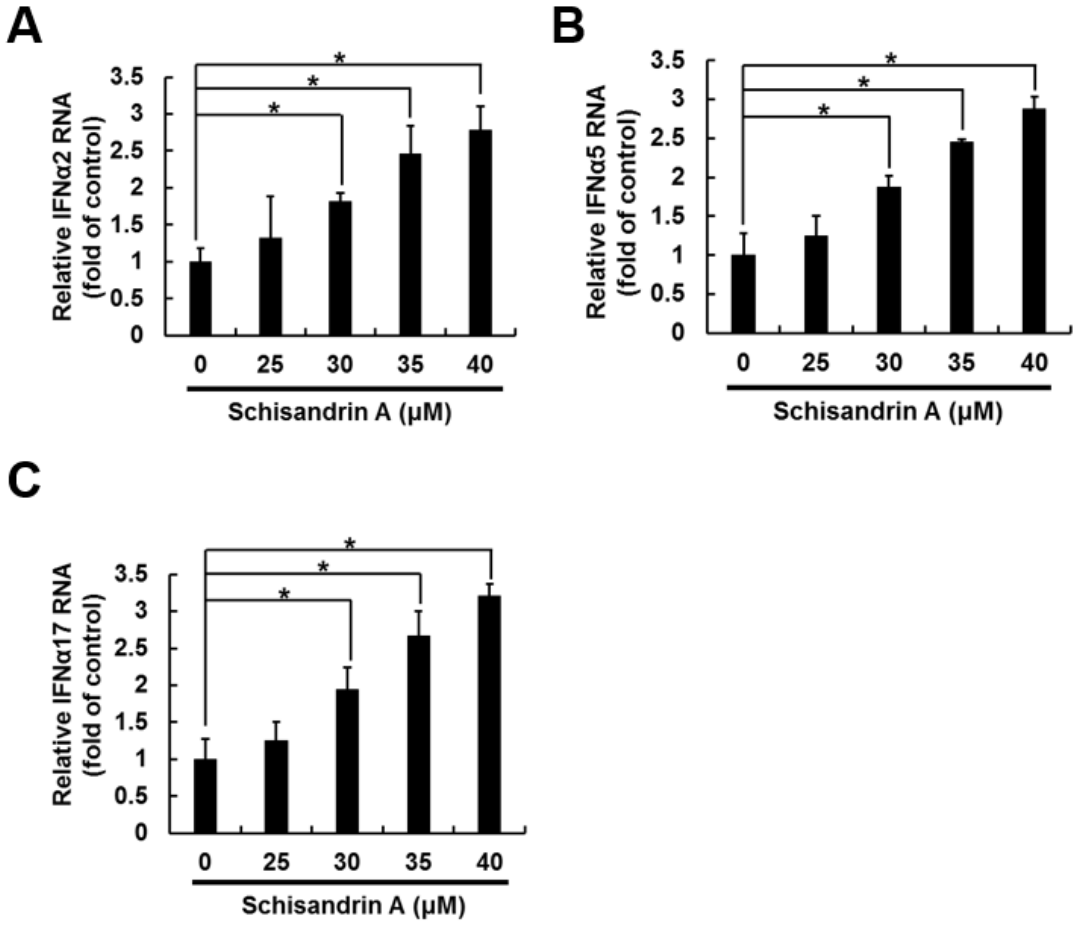

Fig S2. Schisandrin A increases cellular antiviral IFN-α expression in naïve Huh-7 cells.

(A–C) The naïve Huh-7 cells were treated with 0, 25, 30, 35, and 40 μM of schisandrin A for 3 days. Total RNA was collected and the cellular (A) IFN-α-2, (B) IFN-α-5, and (C) IFN-α-17 mRNA levels were determined by RT-qPCR. The gene expression was normalized by the cellular gapdh mRNA level. Error bars denote the means ± SD of five independent experiments (N=5). *P < 0.05.
